# In vitro susceptibility of e.faecalis and c.albicans isolates from apical periodontitis to common antimicrobial agents, antibiotics and antifungal medicaments

**DOI:** 10.4317/jced.50593

**Published:** 2012-02-01

**Authors:** Aysin Dumani, Oguz Yoldas, Sehnaz Yilmaz, Beril Akcimen, Gulsah Seydaoglu, Arzu Kipalev, Fatih Koksal

**Affiliations:** 1 DDS PhD. Assistant Professor, Department of Conservative Dentistry and Endodontics, University of Cukurova, Faculty of Dentistry, Adana, Turkey; 2 DDS PhD. Professor, Department of Conservative Dentistry and Endodontics, University of Cukurova, Faculty of Dentistry, Adana, Turkey; 3 PhD. Research Assistant, Department of Microbiology, University of Cukurova, Adana, Turkey; 4 PhD. Associate Professor, Department of Biostatistic, University of Cukurova, Adana, Turkey; 5 PhD. Professor, Department of Microbiology, University of Cukurova, Adana, Turkey

## Abstract

The aim of this study was to evaluate in vitro antimicrobial activity of 4 antibiotic agents (for E.faecalis) and 4 antifungal agents (for C.albicans) by agar dilution method. Additionally, modified strip diffusion method was used for detection of in vitro antimicrobial activities of 5% NaOCl, 2.5% NaOCl, 17% EDTA and 2% CHX and agar diffusion method for detection of in vitro susceptibilities of three intracanal medicaments for 18 E.faecalis and 18 C.albicans isolates from primary and secondary root canal infection. Isolates were recovered from 231 endodontic samples of patients, with the need of root canal treatment and retreatment. All tested E.faecalis isolates showed resistance to antibiotics. For irrigation solutions, 2% CHX was more effective in eliminating E.faecalis but 5% NaOCl showed larger inhibition zone than 2.5% NaOCl, 17% EDTA and 2% CHX. For intracanal medication, Ca(OH)2-CHX worked efficiently in killing E.faecalis isolates compared to Ca(OH)2-Steril saline solution, Ca(OH)2-Glycerin. For C.albicans, 18 isolates were susceptible to amphotericin B, nistatin, fluconazole but showed resistance to ketoconazole. 5% NaOCl was more effective in eliminating and produced larger inhibition zone compared to 2.5% NaOCl, 17% EDTA and 2% CHX. Ca(OH)2-Glycerin intracanal medication was better in eliminating C.albicans isolates and produced larger inhibition zone compared to other Ca(OH)2 medicaments.

** Key words:**E.faecalis, C.albicans, antimicrobial, antibiotic, antifungal.

## Introduction

The goal of endodontic treatment is the elimination of microbial infection in the root canal system by adequate instrumentation, irrigation and high technical quality of permanent root canal filling. Instrumentation and irrigation provides to remove all necrotic and vital organic tissue, and give the root canal system a shape that allows easy debridement and predictable placement of locally used medicaments. If sufficient instrumentation and irrigation can eliminate all microorganisms in the root canal system at the first appointment, most treatments could be finished in one visit and there will be no coronal leakage of the permanent root canal filling. However, complete elimination of bacteria is not always obtained in clinical practice due to the anatomical complexities of root canals and limitations of medicaments (1).

Recent studies have focused on evaluating the effectiveness of root canal irrigants and medicaments against *Enterococcus faecalis and Candida albicans* (2,3). E.faecalis can grow at pH 9.6 and tolerate pH levels as high as 11.9; so they can withstand harsh enviromental conditions. This makes these bacteria able to survive in the root canal as a single organism or as a major component of the flora (4). *E.faecalis* has been isolated in 23-70% of positive cultures from infected root canals with signs of chronic apical periodontitis (5). In addition, *C.albicans* is the most commonly isolated fungi from oral cavity and the root canal (6).

The ideal irrigating solution must be an antimicrobial agent that does not cause toxic effects to periapical tissues. NaOCl is an effective antimicrobial and organic tissue dissolution agent but at high concentrations, it is highly irritant to the periapical tissues (7). Instrumentation and antibacterial irrigation with NaOCl can eliminate bacteria in 50% to 75% of infected root canals at the end of the first treatment session (8).

Clorhexidine (CHX) gluconate has also been proven to be an effective antimicrobial irrigant but it does not have ability to dissolve the pulp tissue (9). However, Ethylenediamine tetraacetic acid (EDTA) has little antibacterial activity but it has ability to remove the organic portion of the smear layer, so that consequent irrigants could achive dentinal tubules.

Ca(OH)2 has been used as an antimicrobial medicament in dentistry for over 40 years and it has antimicrobial and antifungal activity and tissue-dissolving ability (10). Combinations of Ca(OH)2 with effective disinfectants may provide an improved inter-appointment dressing. If the canal is not dressed with a disinfectant between two visits, microorganisms will multiply rapidly.

The main advantage of local antibiotics compared to systemic use is the prevention of systemic complications and that substantially higher concentrations can be utilized. However, multiple antibiotic resistance in clinical isolates from root canal infections has been reported (11). Attention has been focused on enterococcal susceptibility to different antibiotics since the first report of vancomycin-resistant enterococci (12). Enteorococci have displayed resistance to antimicrobial agents and this resistance may be intrinsic or acquired via gene transferation.

Commonly used antifungal agents may be useful in the treatment of root canal yeast infection, alone or in combination with a disinfectant (13). However, recent research data on antifungal susceptibility of yeasts isolated from root canal infections is scarce.

The aim of this study was (a) to evaluate in vitro activity of four antibiotic and four antifungal agents using the agar dilution method (b) to evaluate the in vitro antimicrobial activity of 5% NaOCl, 2.5% NaOCl, 17% EDTA and 2% CHX solution by strip diffusion method (c) the in vitro susceptibility of three intracanal medicaments by agar diffusion method to *E.faecalis* and *C.albicans* isolates from primary and secondary root canal infection.

## Material and Methods

In the present study 231 endodontic samples of patients requiring root canal treatment or re-treatment who attended to the Cukurova University, Faculty of Dentistry, Adana, Turkey were included. The samples were taken by a general dental practitioner and identification procedures were performed at the Department of Microbiology of the Cukurova University.

The samples were inoculated on Sabouraud’s dextrose agar and blood agar plates and and incubated in aerobic conditions. Suspected colonies of Enterococcus were checked for colonial morphology, Gram-staining, catalase activitiy, 6.5% salt tolerance, oxygen tolerance characteristics and further identification was done by using PCR assay. Identification of *C.albicans* colonies were performed by Gram-staining, germ tube formation testing and carbohydrate assimilation patterns using a commercially available test kit (ATBID 32C, bioMerieux Maray, Etoile, France) and PCR.

A total of 26 *E.faecalis* and 30 *C.albicans* strains were isolated. Eighteen *E.faecalis* and 18 *C.albicans* strains were selected randomly from all obtained cultures. Eleven of the *E.faecalis* strains were isolated from primary apical periodontitis and 7 from cases of secondary apical periodontitis. Twelve of 18 *C.albicans* strains were isolated from primary apical periodontitis and 6 from cases of secondary apical periodontitis.

Susceptibility Test of Antibiotics and Antifungals

The susceptibility tests of 18 *E.faecalis* strains were performed by agar dilution method. The following antibiotics were tested: vancomycin, penicillin, spiramycin and colistin. Antibiotic concentrations were shown in  Table 1.

**Table 1:**
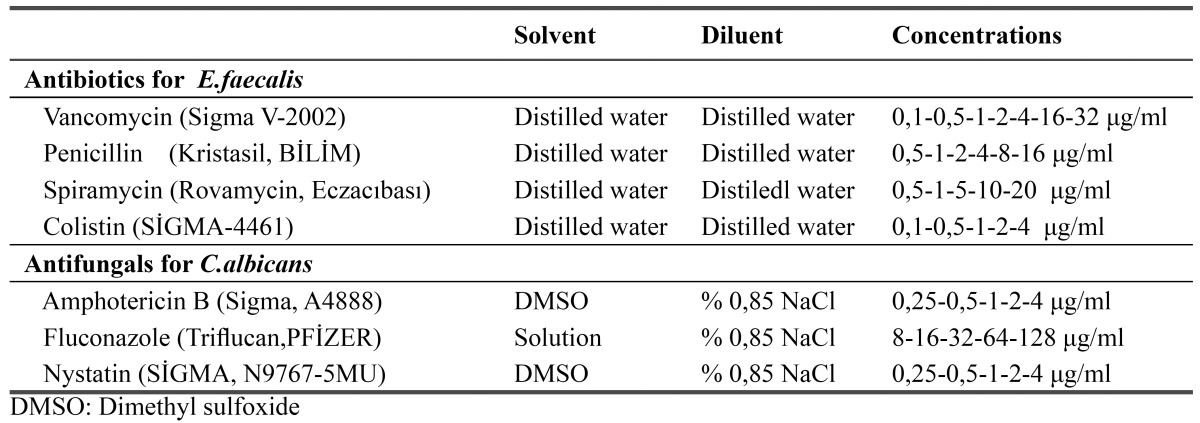
Solvent and dilüent for preparation of stock solution of antimicrobial and antifungal agent.

Pure bacterial strains were inoculated on the Mueller-Hinton agar plates containing different concentrations of antibiotics by using a swab that had been submerged in bacterial suspension standardized to match the turbidity of the 0.5 McFarland standard.

The susceptibility of the 18 clinical *C.albicans* strains to amphotericin B, nystatin, fluconazole, ketoconazole were tested using the agar dilution method. The antifungal agents and yeast inocula were prepared according to M27-A recommendation of the CLSI (Clinical and Laboratory Standards Instıtute). Briefly, amphotericin B, nistatin and ketoconazole were dissolved in dimethyl sulfoxide (DMSO). Antifungal concentrations were showed in  Table 1.

Susceptibility tests of irrigation solutions 

The modified strip diffusion method was used to detect susceptibilities of Enterococcal and Candidal strains to the irrigation solutions of 5% NaOCl, 2.5% NaOCl, 17% EDTA and 2% CHX. Microbial suspensions adjusted to the turbidity of 0.5 McFarland standard were inoculated on Mueller-Hinton and Sabouraud’s dextrose agar with sterile cotton swabs. The surface of the plate was swabbed in three directions to ensure a complete distribution of the inoculum over the entire plate. 0.5x4 cm sized sterile Whatman-3 filter papers were immersed in the antimicrobial suspension (5% NaOCl, 2.5% NaOCl, 17% EDTA, 2% CHX, Sterile saline solution for control) for 5 minutes and after removing the excess fluid, antimicrobial strips were placed on agar plates within 20 minutes of inoculation time. Following inoculation, the plates were incubated at 37 ºC aerobically for 16-18 h. After incubation, zones of growth inhibition were measured around the strips.

Susceptibility tests of intracanal dressing medicaments 

The susceptibility of Enterococcal and Candidal strains to intracanal dressing medicaments were tested by the agar diffusion method. Enterococcal and Candidal suspensions of the strains were prepared with serum physiologic and the turbidity was adjusted to 0.5 McFarland’s turbidity standard. By using sterile cotton swabs, suspensions were used to make a lawn culture of the organisms on Sabouraud’s agar and blood agar plates. Following incubation, four wells with 6 mm diameter and 4 mm depth were punched in each agar plate with a sterile punch. The wells filled with Ca(OH)2 paste prepared with sterile saline solution or Glycerin or 2% Chlorhexidine liquid. Sterile saline solution (140 ml) was used as a control. Cultures were evaluated after 1, 4 and 7 days and the zones of inhibition for each chemical used against a particular isolate were recorded using a transparent ruler.

## Results

All of the E.faecalis strains were resistant to spiramycin and colistin. Nine strains were resistant to penicillin and 10 strains were resistant to vancomycin. Of the strains isolated from secondary endodontic infections, 7 were resistant to spiramycin, colistin and vancomycin but 4 strains were resistant to penicillin.

For *C.albicans* isolates all of the twelve strains from primary endodontic infections were sensitive to nystatin and amphotericin B and were resistant to ketoconazole. Four isolates were intermediate and 8 were sensitive to fluconazole. Of the 6 secondary endodontic isolates, all were sensitive to nystatin and amphotericin B and were resistant to ketoconazole. Two isolates were intermediate and 4 were sensitive to fluconazole.

Irrigating solutions were tested by modified strip diffusion methods. The diameter of inhibition zones were measured and compared. All of the primary endodontic isolates were sensitive to 2% CHX solution while three isolates were resistant to 17% EDTA solution and six were both resistant to 2.5% NaOCl and 5%NaOCl. For inhibition no statistically significant difference was detected between EDTA and 2.5% and 5% NaOCl solutions (p=0.3) and between CHX and EDTA (p=0.2). But a statistically significant difference was found between CHX and 2.5% and 5% NaOCl solutions (p<0.004). 2% CHX was the most effective solution on the inhibition of primary endodontic isolates of E.faecalis. However when 5% NaOCl was effective against isolates, it showed maximum inhibitory zones ( Table 2).

**Table 2:**
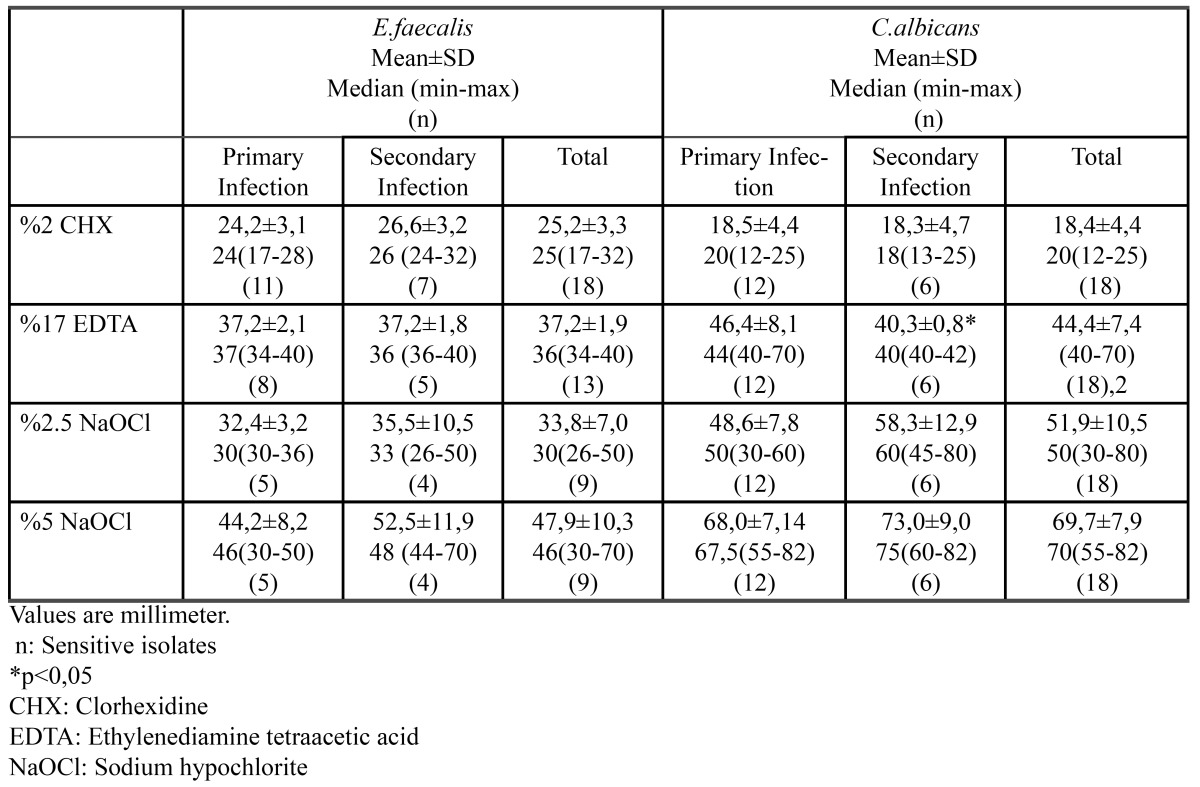
Distribution of inhibition zones of E.faecalis and C.albicans according to irrigation solutions group and type of infection groups.

In secondary endodontic infections, all of 7 isolates were sensitive to CHX solution while 2 isolates were resistant to EDTA and 3 isolates were resistant to both 2.5% NaOCl and 5%NaOCl. CHX showed smaller inhibitory zones than EDTA, 2.5% NaOCl and 5%NaOCl. There was no significant difference between EDTA and 2.5% NaOCl. However 5%NaOCl showed maximum inhibitory zones ( Table 2).

The solutions inhibited the growth of *C.albicans* in all of the primary endodontic isolates. 5% NaOCl revealed maximum efficacy, while 2.5% NaOCl and EDTA were intermediate and 2% CHX was minimum, relatively. The best solution that prevented the growth of *C.albicans* was 5% NaOCl, whilst there was no statistically different between 2.5% NaOCl and EDTA ( Table 2).

Similar results were reported in secondary endodontic infection with primary endodontic infections. 5% NaOCl was the most efficient solution while 2% CHX revealed the least activity compared with the other solutions ( Table 2).

Agar diffusion method was used for measuring suspectibilities of intracanal dressing medicaments. The inhibition zones of each chemical were evaluated at 1, 4 and 7 days. On first day of measurement directed on primary endodontic samples, one of the strains showed no inhibition zone for CHX-Ca(OH)2, 2 strains for Glycerin-Ca(OH)2 and 3 strains for Sterile saline solution-Ca(OH)2. The maximum inhibition zone on the growth of *E.faecalis* strains was detected by CHX-Ca(OH)2. Glycerin-Ca(OH)2 was the second efficient medicament ( Table 3).

**Table 3:**
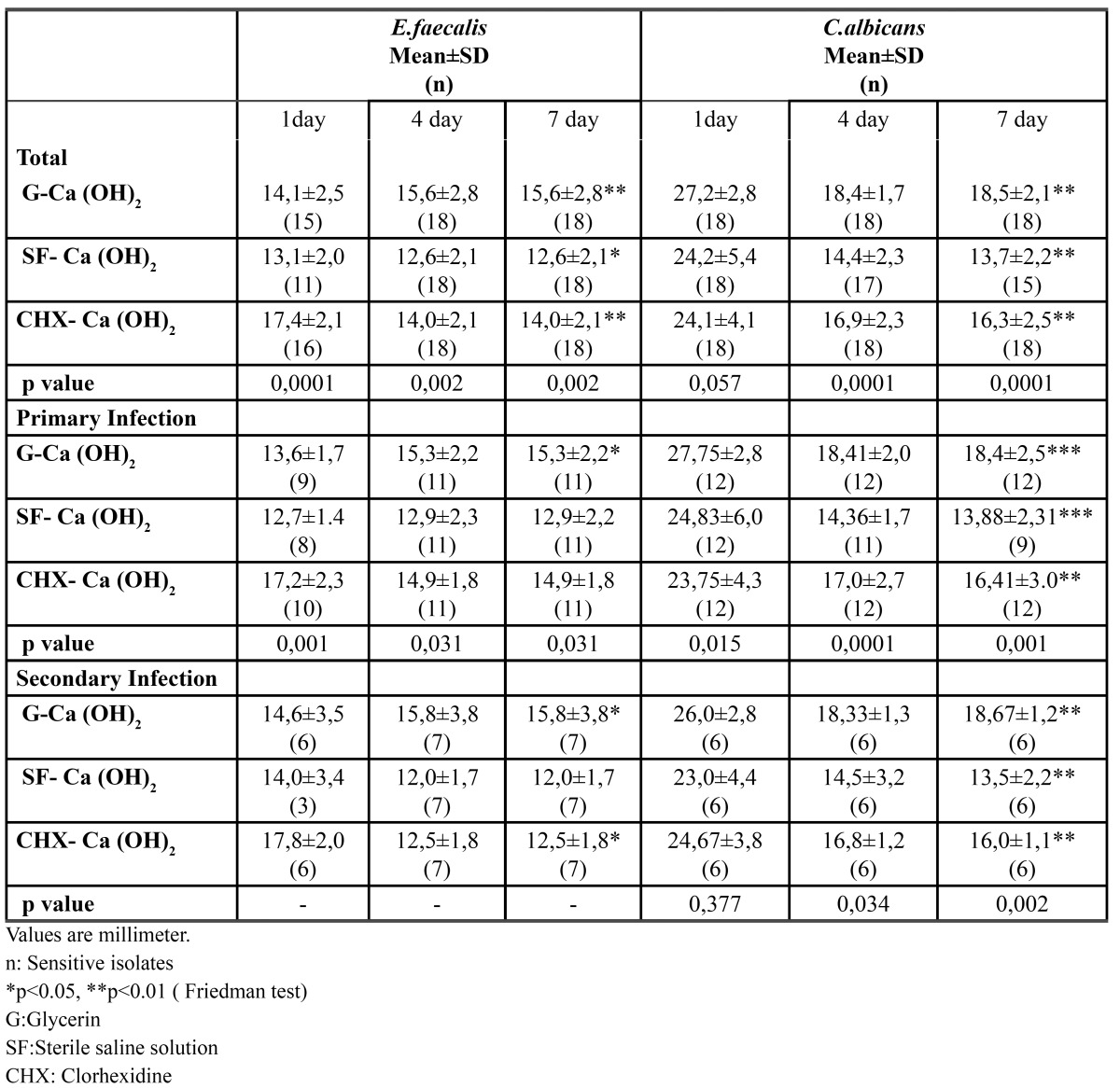
Distribution of inhibition zones of E.faecalis and C.albicans according to intracanal dressing medicaments group and type of infection groups and for time periods.

Of the 7 secondary endodontic samples, one of the strains showed no inhibition zone for CHX-Ca(OH)2 and Glycerin-Ca(OH)2 and 4 strains for Sterile saline solution-Ca(OH)2 at first day. CHX-Ca(OH)2 was the best medicament for the inhibition of the growth of *E.faecalis* and showed maximum inhibition zone in secondary infections ( Table 3).

For *C.albicans* Glycerin-Ca(OH)2 combination showed maximum inhibitor efficacy on primary endodontic isolates on first day. This medicament was statistically better than CHX-Ca(OH)2 and Sterile saline solution-Ca(OH)2. On fourth day, the diameters of inhibition zones were decreased in all of the combinations of Ca(OH)2. There was no statistically significant difference between 4 and 7 day. Sterile saline solution-Ca(OH)2 was not efficient for 1 isolate at 4 day and 2 isolates at 7 day ( Table 3).

The diameters of inhibition zones of secondary endodontic isolates were similar to primary endodontic isolates. Glycerin-Ca(OH)2 medicament showed largest inhibition ( Table 3).

Statistical Analyses

Statistical analysis was performed using the statistical package SPSS v 12.0. For each continuous variable, normality was checked by Shapiro wilks test. Comparisons between groups were applied using the student t test and one way ANOVA test for normally distrubited data and Kruskal Wallis test and Mann Whitney U test were used for the data not normally distrubited. Time dependent intragroup data was analysed using the Friedman test. Wilcoxon rank sum test was used to evaluate the differences within two groups toward the first day values. Bonferroni’s correction was applied (p<0,05/n; n= number of comparisons) when multiple comparison were made. Results were presented as mean± SD and median (min-max).

## Discussion

Endodontists have been aware of the need to use proper antimicrobial strategies that can eliminate fungi and enterococcus from infected root canals (11,13). The aim of the present investigation was to evaluate the antimicrobial effectiveness of endodontic medicaments against *E.faecalis* and *C.albicans* strains that have been detected in primary and secondary root canal infection.

In this study, antibiotics were investigated to aid the host defenses in controlling and eliminating microorganisms that temporarily have overcome the host defense mechanisms (14). However, infections of endodontic origin are treated without antibiotics because there is no blood circulation within a necrotic and infected pulp so antibiotics can’t reach and eliminate microorganisms present in the root canal system (14). And also, antibiotics are not suitable for short-term use as an irrigating solution because of their efficiency during the cell reproductive cycle (15). But use of prophylactic antibiotics in patients at risk for endocarditis should be considered in root canal revision treatment because *E.faecalis* is highly associated with those infections (16).

In this research penicillin, vancomycin, spiramycin and colistin were evaluated by agar dilution method. In previous studies oral enterococci are shown to have high susceptibility to vancomycin (17,18) and showed 10-100 times higher minimum inhibitory concentrations (MICs) against penicillin and ampicillin than other streptococci (18). The presence of enterococcal strains resistant to penicillin has been reported in endodontic infections in the USA(19) and Sweeden (18). Some studies showed a higher susceptibility of *E.faecalis* to benzylpenicillin and vancomycin (11,20). In contrast, Dahlen et al.(18) demonstrated that *E.faecalis* was resistant to benzylpenicillin but sensitive to vancomycin. In this research, five strains of *E.faecalis* were susceptible to penicillin and one strain was susceptible to vancomycin and all 18 strains showed resistance to spiramycin and colistin.

The differences in these studies may be correlated with the type of* E.faecalis* strain, the treatment protocols and the nature of the endodontic infection, geographical differences and changes in resistance pattern of bacteria during time (20).

In the present study, all fungi strains were susceptible to nystatin, amphotericin B and fluconazole but resistant to ketoconazole. In accordance with our study, Kuriyama et al. (21) found almost all of the candidal isolates were found to be susceptible to fluconazole, amphotericin B and nystatin but there has been rising concern about increased resistance of *C.albicans* to azoles (13).

Antifungal susceptibility testing is complex, problematic, and has many inherent difficulties. Hence a standardized technique described by National Committee for Clinical Laboratory Standards (NCCLS) (M27-A) has been advocated for this purpose for generating globally comparable data. To our knowledge there have been very few published studies examining the effect of antimicrobial agents on fungi.

For antimicrobial solutions tested in this study all *E.faecalis* isolates were sensitive to 2% CHX solution but 9 isolates were resistant to 2.5% NaOCl and 5%NaOCl and 3 isolates were resistant to EDTA. There was statistically significant difference between CHX and 5% NaOCl and 2.5% NaOCl for their bactericidal activity. In contrast when inhibitory zones were compared, 5% NaOCl showed maximum inhibitory zones and 2.5% NaOCl and EDTA showed intermediate zones while CHX showed minimum zones. CHX had maximum bactericidal activity but minimum inhibitory zones.

Although there is disparity among experimental designs, in accordance with this study, it was demonstrated that NaOCl did not always eliminate *E.faecalis* and CHX was more effective than NaOCl (1).

In accordance with previously studies, our results showed that EDTA has antimicrobial activity against *E.faecalis* (22,23). However, some authors, especially those using dentin models have reported that EDTA has no inhibitory capacity (24,25). These differences between results may depend on EDTA solutions’ capacity to chelate calcium ions of dentine and become inactive (26).

All irrigation solutions in this study showed antibacterial activity against *C.albicans*. 5% NaOCl revealed maximum efficacy where as 2% CHX showed minimum efficacy and 2.5% NaOCl and EDTA were intermediate. These results are in accordance with previous study (13). However, Sen et al. (27) found that EDTA was the most effective irrigant against *C.albicans* using the agar diffusion test and Ruff et al. (2) reported, 6% NaOCl and 2% CHX were equal.

*E.faecalis* is resistant to Ca(OH)2 both in vivo and in vitro. *E.faecalis* probably has an effective proton pump mechanism which maintains optimal cytoplasmic pH levels. Evans et al.(28) demonstrated that CHX combined with Ca(OH)2 will result in a greater ability to kill *E.faecalis*. In our study CHX-Ca(OH)2 combination was effective in shorter periods of time. But at day 7 there was no significant differences among Ca(OH)2 combinations.

 In our study *C.albicans* was most sensitive to Glycerin-Ca(OH)2 in all time periods. However Siqueira et al.(29) reported that Glycerin-Ca(OH)2 combination was effective after 7 days of exposure but the CHX-Ca(OH)2 combination was ineffective in disinfecting dentin even after 1 week of medicament exposure. *C.albicans* is an alkali-resistant microbe so in previous study resistance to Ca(OH)2 combinations were demonstrated (29).

The differences between these studies and the present investigation may have been caused by different experimental methods. In this study for antibiotics and antifungals agar dilution method, for dressing material agar diffusion method was prefered. Dilution method gives a quantitative result for the amount of antimicrobial agent that is needed. But it is time consuming and can be only used with substances that are soluble in the culture medium. Agar diffusion method gives an inhibition zone around the discs containing the agent and direct exposure method provides qualitative information about the substance (3). But the size of the microbial inhibition zone depends upon the solubility and diffusibility of the test substance and so may not express its effective full potential. And also solid medium would make the diffusion of hydroxyl ions in Ca(OH)2 pastes more difficult (3). In agar diffusion tests, the pH of the substrate, incubation period, and toxicity, sensitivity, and particularly diffusion of the drug may have an impact on the antimicrobial activity of the test materials. Modified strip diffusion method was used for irrigating solutions. This method is correlated to substance effectiveness and its direct contact with microorganisms (3).

The collection of the strains took about 2 years and within the limitations of this study following conclusions were drawn. (a) There is no statistical difference between the resistance pattern of *E.faecalis* and *C.albicans* isolated from primary and secondary root canal infections to selected antimicrobial agents; (b) For *E.faecalis* isolates while the highest ratio of inhibition was detected in CHX group, the lowest mean of inhibition zones were found in this group comparing other solutions groups. Controversially, the lowest ratio of inhibition was detected in NaOCl group, the highest mean of inhibition zones were found for this group so combined use with 2% CHX is recommended; (c) All irrigation solutions were effective in eliminating *C.albicans* isolate; (d) CHX-Ca(OH)2 combination showed maximum efficacy for elimination of *E.faecalis* where as Glycer-in-Ca(OH)2 combination was effective for elimination of *C.albicans* isolates.
